# Green Biosynthesis of Silver Nanoparticles Using *Malva parviflora* Extract for Improving a New Nutrition Formula of a Hydroponic System

**DOI:** 10.1155/2022/4894642

**Published:** 2022-05-30

**Authors:** A. G. Oraibi, H. N. Yahia, K. H. Alobaidi

**Affiliations:** Department of Plant Biotechnology, College of Biotechnology, Al-Nahrain University, Baghdad, Iraq

## Abstract

There are increasing needs for developing nontoxic, low-cost, high-yield, and eco-friendly procedures for manufacturing nanoparticles. Nanobiotechnology can be used in food security for improving crop production; nanoparticles could enhance the growth and yield of different crop plants; therefore, this work aimed to improve a new nutrition formula of a hydroponic system using green biosynthesis of silver nanoparticles and *Malva parviflora* aqueous extract. Results shown that AFM image of AgNP surface morphology provides good indicator for biosynthesizing AgNPs. UV-vis spectroscopy showed the presence of silver elements that proved the reduction of silver ion to an element in the presence of plant extract functional groups which act as a reduction reaction capping agent. AgNPs formation from 1 mM of AgNo_3_ and *Malva parviflora* filtrate can easily be characterized through visual observations by the change in the color of the reaction mixture from green to yellowish-brown. SEM showed that most of the Ag nanoparticles were spherical in shape, well dispersed, and were either arranged in clusters of particles with each other, or as small particles, and have been identified in a size range of 12–63 nm. The EDX characterization exhibited that the highest proportion of the element composition was for silver weighting (34.11%) in nanoparticle. Other elements such as aluminum (12.28%), carbon (8.62%), hafnium (18.12%), nitrogen (9.34%), sodium (10.01%), and oxygen (7.52%) may arise from *Malva parviflora* extract. Also, peroxidase and catalase enzyme activity, cabbage crop seedlings, fresh and dry weights, and proline and carbohydrate concentrations were significantly increased with the increase of biosynthesized AgNP concentrations but up to limit.

## 1. Introduction

Green biosynthesis of AgNPs using different parts of plants has attracted great attention in industrial and medicinal applications. AgNPs were *in vitro* green biosynthesized using *A. graecorum,* and its antitumor and anti-fungal activities were studied. SEM (scanning electron microscopy) image result indicated a spherical shape of AgNPs with 22–36 nm size range. FTIR (Fourier transform infrared spectroscopy) exhibited the functional groups that represented the groups involved in the reduction of silver ion into nanoparticles and concluded that the synthesized silver nanoparticles from *A. graecorum* can be used as a potential antifungal and antitumor agent for various therapeutical applications [1].

*Malva parviflora* L. (cheeseweed mallow) was used for green synthesis of AgNPs, and the AgNPs were detected using UV-vis spectroscopy and FTIR (Fourier transform infrared spectroscopy). Also, transmission electron microscopy (TEM) was used to characterize the size and shape distribution, zeta potential analysis, and FE-SEM (field emission scanning electron microscopy). The *M. parviflora* leaf extract chemical composition was identified using mass spectroscopy and gas chromatography (GC/MS) [2].

Hydroponics is a convenient method for studying plant growth and development *in vitro*. It is a useful technique to grow plants under controlled nutritional conditions. It is an agricultural technique to grow plants in nutrient solution with complete absence of soil. Soil was substituted by sterile culture mediums such as rockwool, vermiculite, sand, gravel, clay pellets, and perlite to give stability to the roots. Nutrients were passed through roots differently, depending on the type of the hydroponic system that was used. Oxygen was pumped through, pH level was regulated, and sufficient light was provided to carry out photosynthesis [3]. With the world's growing population, the global food demand increased, providing a new sustainable agricultural method. Microgreen and soilless farming combined with green nanotechnology provide a revolutionary solution and are more sustainable to conventional farming. There are some applications of nanotechnology in microgreen and soilless farming that include (1)improvement of plant traits against stress and environmental diseases through nanomaterial application; (2) improvement the plant tissue or organelle function through plant nanobionics; and (3) the extending the life of delicate vegetables using bio-impregnated nanoparticles on the packaging or by using any other methods for preservation [4]. Cabbage crop is a crop with high nutritional values and an excellent source of folic acid which has importance in the formation of blood cells, vit A, vit B, and calcium. Also, it provides vit C and mineral salts such as potassium, magnesium, sodium, and calcium. It is excessively use in human food, with a high level of consumption that reached approximately 500 g/person/day [5]. This study aimed to improve a new nutrition formula of a hydroponic system using green synthesis of silver nanoparticles using *Malva parviflora* plant aqueous extract.

## 2. Materials and Methods

### 2.1. Preparation of *M. parviflora* Watery Extract

Aqueous extract of *M. parviflora* plant was prepared according to the following procedure: plant leaves were cleaned well with running water, soaked for 30 min, dried well using dry air, cut in to small pieces using sterilized scissors, mashed well, and then weighed 100 gm. The mashed explants then were macerated in a 500 ml glass beaker containing sterile ddH_2_O (Ajax, Australia) for seven days and then filtered using a Whatman No.1 filter paper (Millipore, England).The final extract was then stored at 4°C in a refrigerator for later use [6].

### 2.2. Plant-Based Synthesis of AgNPs (Green Manufacture of Silver Nanoparticles)

Silver nanoparticles were green-synthesized from the aqueous extract of *M. parviflora* plant leaves by mixing 10 ml of the watery plant extract with 100 ml of 1 mM of AgNO_3_ and stirred using a hot plate-magnetic stirrer (Gallenkamp, England) at 45°C for 15–20 min in the dark. The biosynthesized AgNPs were purified using the repeated centrifugation method (Daihan, Korea) at 12,000 rpm for 20 min followed by the redispersion of pellet in the sterilized DDWH_2_O (Ajax, Australia). A gray precipitate was collected by filtration. The crystal form of green-synthesized AgNPs were then used as powder phase in the characterization of the manufactured AgNPs, and 100 ml of 1 mM of AgNO_3_ was kept at as control [7].

### 2.3. Characterization of the Green-Manufactured AgNPs

#### 2.3.1. A Visual Observation

The process of metal-ions reduction during the reaction can be detected by converting the pale-yellow color of the mixture into a greenish-yellow solution.

#### 2.3.2. SEM (Scanning Electron Microscopy)

Sample analysis using a SEM (MIRA3, France) examination was used for diagnosis and characterization of the green-manufactured AgNPs through the determination of the size and shape of the particles in the tested samples [8]. These examinations were done in the physics department laboratory, College of Science, Al-Nahrain University.

#### 2.3.3. Atomic Force Microscopy (AFM)

AFM analysis was used to diagnosis the morphology of the manufactured AgNPs surface. It was done under normal atmospheric conditions using Angstrom advanced (AA2000) scanning prop microscopy (NT-MDT, Russia). The solution of the samples (AgNPs) was diluted with DDWH_2_O, and then, small drops of these samples were placed on a microscope glass slide (1 cm × 1 cm) and left to be dried. The slide was then placed on the sample-stage of the AFM instrument [8].

#### 2.3.4. UV-Visible Spectroscopy

The manufacturing of AgNPs from silver ions and plant extract was monitored using UV-Visible spectra by using respective solutions diluted for 20 times with sterilized distilled water. Spectrum was recorded using UV-1700, Shimadzu, Japan, from 100 nm–550 nm. The blank was sterilized with distilled water.

### 2.4. Preparation of Biosynthesized Silver Nanoparticle Concentrations

Different concentrations of green-manufactured silver nanoparticles were prepared (0.0, 0.5, 1.0, 2.5, 4.0, and 5.5 mg/ml) by dissolving a specific weight of the biosynthesized nanoparticle powder in sterilized distilled water.

### 2.5. Preparation of a Hydroponic System

A hydroponic system was prepared comprising 42 plots. Each one represented one nutrient film technique-independent hydroponic system. This system was a constituent from the hydroponic profiles, i.e., polypropylene with a diameter of 75 mm, 2 m length, holes were of 2 mm, with 0.285 m spacing between the plants and 0.25 m between the profiles, with seven plants in each profile. These profiles were installed at 0.75 m height, and the injected solution was run down through the profile slope at a 1.0 L/min average rate; a stop cock was used for regulating the flow. Each 100 L of the nutrient solution used in the hydroponic system contained 1.0 L of A solution (12 g Fe^++^ and 200 g Ca) and 1.0 L of B solution (20 g Mg, 28 g N P.K and 8 g micronutrients). Cabbage seeds were placed in to 2 × 2 × 2 cm cells of the phenolic foam for germination, with 3 seeds/cell. After sowing, the plates of the phenolic foam were kept in darkness for 7 days. The seedlings were then transferred to hydroponic profiles, submitted to the above nutrient solutions and treated with the different concentrations of biosynthesized silver nanoparticles three times weekly (for four weeks) using foliar spray [5].

### 2.6. Estimation of Catalase Activity (Unit: mg Protein^−1^)

The activity of the catalase enzyme was measured by using hydrogen peroxide disappearance through a spectrophotometer instrument with 240 nm wavelength. The solution used was phosphite buffer (Vivantis, Germany) solution with 0.05 molarity at 7pH; which was prepared by dissolving one pill of ready made buffer solution in 100ml DDWH_2_O and a 30 ml hydrogen peroxide (BDH, England) solution which has been prepared by diluting 50% hydrogen peroxide (0.17 mL) into 100 mL of buffered phosphite. 0.4 mL hydrogen peroxide was mixed with 2.5 mL regulated phosphite and placed in a spectrophotometer (Shimadzu, Japan) for 30 sec, and 0.1 mL of the prepared sample was added.The decreased absorption amount was measured at 240 nm wavelength in 60 s [9].

### 2.7. Estimation of Peroxidase Enzyme Activity (Unit: mg Protein^−1^)

The activity of peroxidase was estimated by measuring the rate of hydrogen peroxide decomposition by enzyme POD with Quinol HCl, determined through measure the color change rate using a spectrophotometer in 470 nm wavelength. The solutions used in this measurement are 7 pH phosphite buffer solution, which was prepared using Quicol by dissolving 11.5 mg dye powder in 5 ml sterilized DDWH_2_O, hydrogen peroxide, which was prepared by dissolving 0.6 ml hydrogen peroxide at 50% concentration in 100 ml of sterilized DDWH_2_O. The mixture of the reaction consisted of 2.9 mL of solution, 0.05 ml of alcohol, 0.05 ml of hydrogen peroxide, and 0.1 ml of enzyme extract, and the rate of light absorption increasing at 470 nm wavelength was recorded by a spectrophotometer (Shimadzu,Japan).

### 2.8. Determination of Fresh and Dry Weights

Seedlings, fresh or dry weights (mg/plant), were measured using a sensitive balance (Denver, Germany), plant tissues were dried in room temperature for 14 days, and dry weights were also recorded using a sensitive balance.

### 2.9. Determination of Proline Continent (Unit: *µ*g·gm^−1^)

Dried plant tissues, about 25 mg dry weight, were homogenized using 3% sulfosalicylic acid (BDH, England) The filtrate was mixed with 5 ml of a ninhydrin reagent and glacial acetic acid (BDH, England), incubated at 100°C for 30 min, and then mixed with 4 ml/toluene (BDH, England). The light absorption of toluene phase was measured at 520 nm using a spectrophotometer instrument [10].

#### 2.9.1. Preparation of Proline Standard Curve

The proline standard curve was plotted using different concentrations of proline 0, 0.5, 1.5, 3.0, 5.0, 7.5, 9.0, 10.5, and 12.0 mg/ml. 5 ml of glacial acetic acid and ninhydrin reagent (BDH, England) were added to all the prepared proline concentration and then incubated for 30 min at 100°C. Samples were then mixed with 4 ml toluene (BDH, England), and light absorption of the toluene phase was estimated at 520 nm using a spectrophotometer.

### 2.10. Determination of Soluble Carbohydrate %

Carbohydrate concentrations were determined based on the phenol sulfuric acid method.25 mg of the dried tissues were homogenized with sterilized distilled water (Ajax, Australia) The filtrate was treated with 3 ml of 5% phenol and 3 ml of 98%sulfuric acid (BDH, England); then, the mixture was incubated for 20 min at 30°C, and absorbance at 485 nm was determined by a spectrophotometer [11].

#### 2.10.1. Preparation of Glucose Standard Curve

The glucose standard curve was drawn by preparing the following glucose concentrations 0.0, 10, 20, 40, 60, 80, and 100 mg/ml. 5 ml was taken from each concentration and treated with 3 ml of 5% phenol and 3 ml of 98% sulfuric acid (BDH/England). The mixture was incubated for 20 min at 30°C,and absorbance at 485 nm was estimated by a spectrophotometer.

### 2.11. Experimental Design and Statistical Analysis

IBM SPSS Statistics Base was used in data analysis as a factorial experiment. It was a completely randomized blocks design (CRBD) with 7 replications for each concentration and treatment at *p*=0.05.

## 3. Results and Discussion

### 3.1. Detection and Characterization of Green-Synthesized AgNPs Using AFM

**Figure 1:**
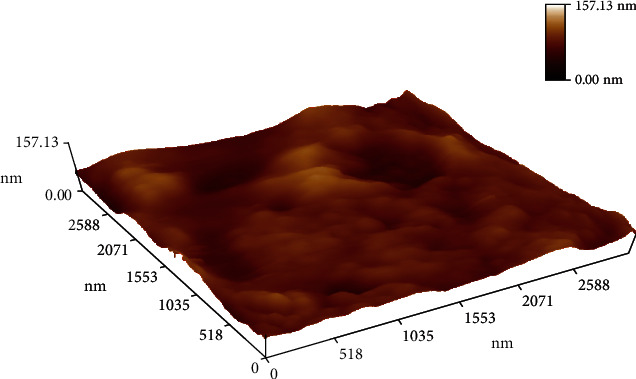
AFM of biosynthesized silver nanoparticles.

**Figure 2:**
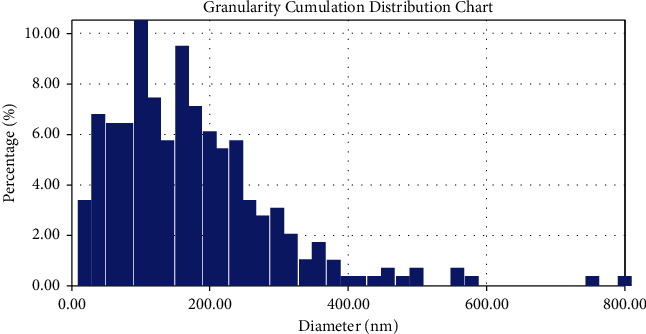
Granularity cumulating distribution chart.

**Figure 3:**
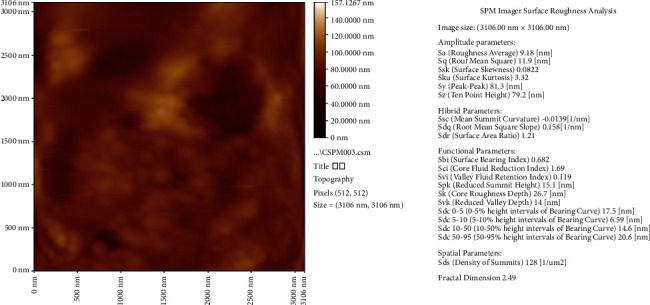
AFM image surface roughness analysis.

The AFM image of the AgNPs film gives a good indicator for the reduction of silver nitrate to silver nanoparticles. The average particle size that was determined in the AFM image was about 157.13 nm as shown in Figure 1. The results are obtained from the AFM measurement shown in the form of a histogram of AgNPs percentages as a function of grain size as shown in Figure 2, 3 and Table 1 By the electro-chemical deposition method, AgNPs particles have been deposited in separated forms and groups of nanoparticles. But the big sizes of nanoparticles shown in AFM analysis occurred because of coagulation of small nanoparticles.

Surface topology of formulated biosynthesized AgNPs was characterized by AFM analysis that showed the topographic structures in 2D or 3D. The types that seem to have very smooth surfaces with grains nearly equal to the starting nanopowder have the sizes measured in the range of 102, 112, and 114  nm for 1^*∗*^10^−3^, 1^*∗*^10^−4^, and 1^*∗*^10^−5^ M aqueous AgNO_3_ solution with *M. parviflora* extract [12].

### 3.2. Characterization of AgNPs by Visual Observation and UV-Visible Spectroscopy

**Figure 4:**
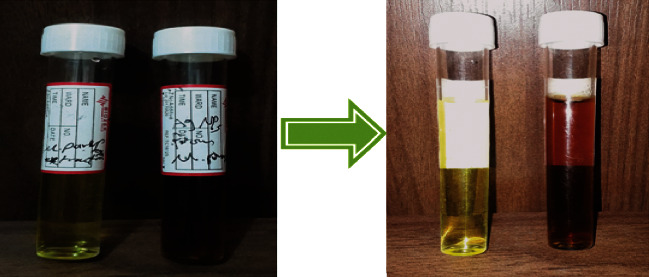
Color change during the reduction process for biosynthesis of AgNPs.

Biosynthesizing of AgNPs from 1  mM of AgNO_3_ solution with *M. parviflora* extract filtrate can be recognized easily by change in mixture color from green to yellowish-brown color as shown in Figure 4. This color arises by the excitation of the surface plasmon vibration in the metal nanoparticle. These results were in line with a study conducted by Abdullah and Dhahi [13] that dealt with green AgNPs manufacture using the seed aqueous extract of grape plant.

**Figure 5:**
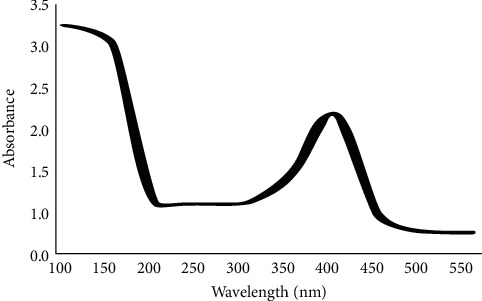
The absorbance of biosynthesized AgNPs using UV-vis spectrum.

Also, the results in Figure 5 show the presence of silver elements that proved the reduction of silver ions to element in the presence of plant extract functional groups which act as a capping agent in the reduction reaction. A visible range of 400–500 nm was typical for AgNPs absorption because they having *λ* max values in this range. Absorption peaks of biosynthesized AgNPs were obtained around 400–420 nm, so these results revealed the successful reduction of Ag + ions to AgNPs in aqueous AgNO_3_ and the biosynthesis of AgNPs. During the short time, the broad spectrum formed was an indication for the presence of nanoparticles in a broad-size distribution. Farhan *et al.* also showed that UV-vis spectroscopy is one of the most widely used techniques for structural characterization of silver nanoparticles [12]. UV-vis spectrum was the first technique used for characterization of metallic nanoparticles [13]. Agudelo *et al.* [14] reported that a Shimadzu UV-1800 UV-visible spectrophotometer was used for analyzing the synthesized gold and silver nanoparticles in a range from 300 to 800 nm; the reducing agent solutions without the precursor agent were used as control. Also, UV-vis spectroscopy represents a convenient technique for characterizing the nanomaterials, as it allows fast data acquisition, and it is available in most chemistry laboratories. This technique can, in theory, be used for the characterization of plasmonic nanomaterials synthesis kinetics [15].

### 3.3. Characterization of Biosynthesized AgNPs Using SEM-EDX Analysis

**Figure 6:**
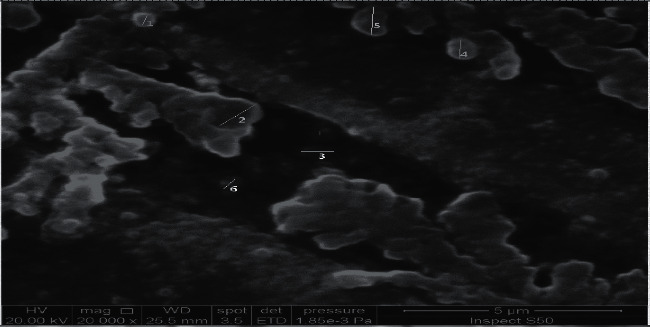
SEM analysis of green-synthesized AgNPs by *Malva parviflora* leaves extract.

The surface topography of the green-biosynthesized AgNPs was determined using a scanning electron microscope (SEM) with a magnification area of 5 micrometers and a magnification force of ×20000. A SEM apparatus was used for the examination of the surface of the adsorbent. Results observed in Figure 6 and Table 2 shown that the biosynthesized AgNPs were spherical-shaped and well dispersed. Also, they are either arranged in clusters of particles with each other, or as small particles, and have been identified in size ranges of 12–63 nm. Farhan *et al.* reported that SEM provides an insight into morphology and size properties of AgNPs [12].

**Figure 7:**
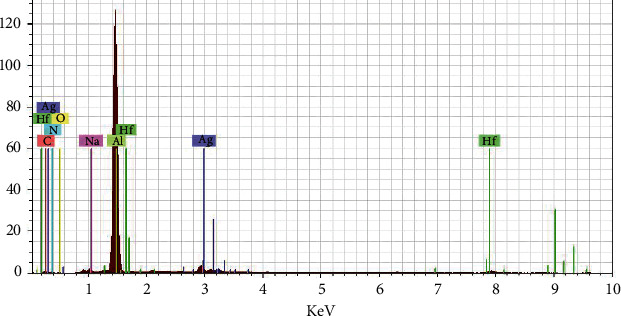
EDX analysis of green-synthesized AgNPs by *Malva parviflora* leaves extract.

EDX analysis gave qualitative and quantitative properties of the full contents of the synthesized nanoparticle.  Figure 7 shows the elemental profile of synthesized AgNPs using the extract of *Malva parviflora* leaves. Also, the results in Table 3 show that the EDX-element percentage contents of biosynthesized AgNPs from plant leaves extracts exhibited that silver provides the highest weight proportion recording (34.11%) in nanoparticle. Other elements such as aluminum (12.28%), carbon (8.62%), hafnium (18.12%), nitrogen (9.34%), sodium (10.01%), and oxygen (7.52%) may arise from the *Malva parviflora* leaves extract, and these results were in line with those obtained by Scimeca *et al.* [16] who revealed that EDX analysis could be also used for the element types (qualitative) as well as for the concentration percentage of each sample element (quantitative) analysis. Also, Iqbal *et al.* [17] reported that energy-dispersive X-ray spectroscopy and SEM were employed to characterize the surface morphology and the composition of the constituently elements.

Dada *et al.* [18] also reported the biosynthesizing of AgNPs from *Calotropis procera*, and these particles were diagnosed through different techniques such as UV-vis spectroscopy, FTIR, SEM,TEM, and EDX. The UV-vis recorded 420 nm as a wavelength for the biosynthesized AgNPs. Green-synthesized silver nanoparticles were also characterized using FTIR (Fourier-transform infrared spectroscopy), XRD (X-ray diffraction), and HRTEM (high-resolution transmission electron microscopy) for characterization or determination of the particles size or morphology. In XRD analysis, the average particle size was found to be 18.31 nm, and TEM analysis showed a face-centered cubic structure with crystalline morphology [19].

### 3.4. Green-Manufactured AgNP Effects on Catalase and Peroxidase Activity of Cabbage Crop Cultured in the Hydroponic System

**Figure 8:**
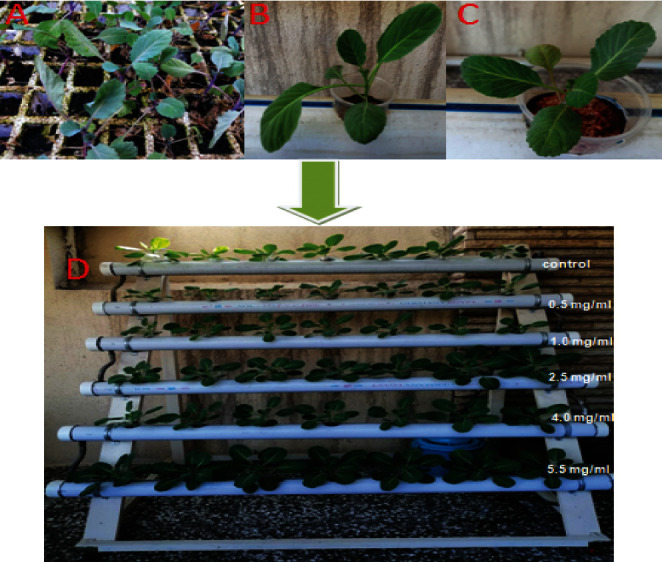
Cabbage crop cultures in the hydroponic system: (a) in the first week of culture; (b) in the second week of culture; (c) in the third week of culture; (d) in the fourth week of culture, *n* = 7.

Results in Figure 8 show the effects of AgNP concentrations on a cabbage plant grown in the hydroponic system, while data in Table 4 reveal that the enzyme activity was significantly affected by the treatment of cabbage crop seedlings with biosynthesized AgNPs. The highest value of the catalase activity was obtained in 4.0 mg/ml AgNPs recording 0.89 mg protein^−1^, also, the peroxidase enzyme activity increased significantly in 2.5 and 4.0 mg/ml AgNPs recording 2.69 and 2.71 mg protein^−1^, respectively, compared to the control (1.52 mg protein^−1^) . These results were in agreement with those obtained by An *et al.* [20], who investigated the positive effect of cerium oxide NPs, showing a significant decrease in ROS levels and an increase in the calcium content in treated plants, and the NPs have been shown to affect ROS and Ca^2+^-mediated signaling genes. Also, Ca^2+^ and ROS are important factors and are effective in the response to stress in plants. Terpene synthetase genes (CAD1 and TPS) are also affected by cerium NPs.

Ali *et al.* [21] exhibited that enzymes activities such as superoxide dismutase “SOD/4.8 U/mg,” peroxidase “POD/3.3 U/mg,” catalase “CAT/2.5 U/mg,” and ascorbate peroxidase “APX/1.9 U/mg” were obtained in the higher level of “90 *μ*g/l” the biosynthesized AgNPs studied for culture proliferations *in vitro*. The AgNPs application also increased the antioxidant enzymes activity, such as CAT, SOD, and POD enzymes, and the expression or activity of CHS or PAL enzymes increased significantly under the treatment of AgNPs. The results exhibited that increasing the antioxidant enzymes activity and regulating the expression and activity of CHS or PAL enzymes are mechanisms to counteract the oxidative stress induced by AgNP treatment in the purslane plants [22].

### 3.5. Effect of Biosynthesized AgNPs on Fresh Weight, Dry Weight, Proline, and Carbohydrate Concentrations of Cabbage Crop Seedlings Cultured in the Hydroponic System

Results shown in Table 5 and Figure 8 reveal that fresh and dry weights increased significantly with the increase of biosynthesized AgNP concentrations, and the highest values for the cabbage seedlings fresh weight were 2.5, 4.0, and 5.5 mg/ml in AgNPs recording 7.51, 7.87, and 5.93 g, respectively. However, cabbage seedlings' dry weight increased significantly in 2.5 and 4.4 mg/ml in AgNPs recording 0.84 and 0.92 mg, respectively, in comparison with control (0.46 mg). Also, the proline and carbohydrate concentration increased significantly with the increase of biosynthesized AgNP concentrations; the highest proline concentrations were 4.0 and 5.5 mg/ml AgNPs (7.41 and 6.93 *µ*g·gm ^−1^ fresh weight, respectively) compared to control (1.38 *µ*g·gm^−1^ fresh weight), but the carbohydrates concentration increased significantly from 5.5 mg/ml AgNPs recording 73.62 *µ*g·gm^−1^ fresh weight compared to control 38.82 *µ*g·gm^−1^ fresh weight as shown in Table 6.These results were in line with those obtained by Mohamed *et al.* who reported that seed treated with synthesized AgNPs increasing in shoot fresh and dry weights were occurred. While decreasing obtained in the total soluble sugars and proline compositions when seeds were treated with low concentrations of synthesized AgNPs and these contents increased significantly in the higher Ag NPs concentrations compared to the control [23]. Also, Mehmood and Murtaza [24] exhibited that biochemical analysis of seeds showed that plants treated with manufactured AgNPs had higher seed carbohydrate or protein concentration, leading to an improvement in the growth and yield. However, Al-Huqail *et al.* [25] revealed that significant decreasing in shoot or roots elongation, shoot or roots fresh weights and the total chlorophyll, total protein contents also studied under the treatment with higher concentrations of biosynthesized AgNPs.

A significant increase was observed in the leaf total chlorophyll contents. The % of total protein or soluble carbohydrate in the shoots and grain increased with an increase in green-manufactured AgNPs concentrations, and significant differences in soluble carbohydrate % was exhibited between the two studied cultivars in grains, catalase, or peroxidase activity, significantly affected in the two studied cultivars, that the highest catalase activity level diagnosed in 1.0 and 1.5 mg/ml (0.68 and 0.69 *µ*g·gm^−1^ fresh weight) in comparison to the +ve control (0.61 mg protein^−1^) [26].

## 4. Conclusion

*M. parviflora* aqueous extract had proven its efficiency in the green-synthesis of silver nanoparticles that had been revealed and characterized using different physical and chemical techniques, such as AFM, UV-vis spectroscopy, SEM, and EDX. Results indicated the green manufacturing of silver nanoparticles according to the nanoparticle size, the particles' dimensional shapes, the proportion of element compositions, and other properties of the nanoparticles that were revealed using the aforementioned techniques. Biosynthesized silver nanoparticles that were manufactured from the plant aqueous extract showed their effectiveness in increasing the enzymatic activity of peroxidase and catalase enzymes, as well as increasing the fresh weight, dry weight, and proline and carbohydrates concentrations of the cabbage crop seedlings that were grown in the hydroponic system.

## Figures and Tables

**Table 1:** Granulation distribution reports.

Average diameter: 164.57(nm)			<=10% diameter: 20.00(nm)					
<=50% diameter: 140.00 (nm)			<=90% diameter: 300.00 (nm)					
Diameter (nm)	Volume (%)	Cumulation (%)	Diameter (nm)	Volume (%)	Cumulation (%)	Diameter (nm)	Volume (%)	Cumulation (%)

20.00	3.39	3.39	220.00	5.42	74.92	420.00	0.34	96.27
40.00	6.78	10.17	240.00	5.76	80.68	440.00	0.34	96.61
60.00	6.44	16.61	260.00	3.39	84.07	460.00	0.68	97.29
80.00	6.44	23.05	280.00	2.71	86.78	480.00	0.34	97.63
100.00	10.51	33.56	300.00	3.05	89.83	500.00	0.68	98.31
120.00	7.46	41.02	320.00	2.03	91.86	560.00	0.68	98.98
140.00	5.76	46.78	340.00	1.02	92.88	580.00	0.34	99.32
160.00	9.49	56.27	360.00	1.69	94.58	760.00	0.34	99.66
180.00	7.12	63.39	380.00	1.02	95.59	800.00	0.34	100.00
200.00	6.10	69.49	400.00	0.34	95.93			

**Table 2:** Particle size of green-synthesized AgNPs by *Malva parviflora* leaves extract using SEM.

Number of lines	Particle size (nm)
1	12.2
2	30.5
3	10.2
4	43.0
5	21.2
6	63.0

**Table 3:** EDX analysis for the elemental percentage contents of biosynthesized AgNPs from *M. parviflora* leaves extract.

Elements	Atomic number	Weight ratio
Aluminium	13	12.28
Carbon	6	8.62
Hafnium	72	18.12
Silver	47	34.11
Nitrogen	7	9.34
Sodium	11	10.01
Oxygen	8	7.52
		100

**Table 4:** AgNP effects on catalase or peroxidase enzymes activity, *n* = 7.

Type of enzyme	Green-manufactured AgNPs concentrations (mg/ml)					
	0.0	0.5	1.0	2.5	4.0	5.5
Catalase	0.39	0.35	0.61	0.53	0.89	0.42
Peroxidase	1.52	1.29	1.31	2.69	2.71	1.94
LSD 0.05	Catalase = 0.36			Peroxidase = 1.21		

**Table 5:** Effect of green-synthesized AgNPs on plant fresh and dry weights (mg), *n* = 7.

Cultivars	Green-synthesized AgNPs concentrations (mg/ml)					
	0.0	0.5	1.0	2.5	4.0	5.5
*Fresh weight*	3.16	2.33	2.84	7.51	7.87	5.93
Dry weight	0.46	0.31	0.41	0.84	0.92	0.36
LSD 0.05	Fresh weight = 2.61			Dry weight = 0.33		

**Table 6:** Green-manufactured AgNP effects on proline and carbohydrate concentrations, *n* = 7.

Parameter	Biosynthesized silver nanoparticle concentrations (mg/ml)					
	0.0	0.5	1.0	2.5	4.0	5.5
Proline	1.38	3.46	1.22	3.82	7.41	6.93
Carbohydrate	38.82	26.81	35.96	29.31	52.01	73.62
LSD 0.05	Proline = 5.31;			carbohydrate = 38.62		

## Data Availability

Data are available upon request from the corresponding author.
